# Research progress of chilled meat freshness detection based on nanozyme sensing systems

**DOI:** 10.1016/j.fochx.2024.101364

**Published:** 2024-04-08

**Authors:** Guangchun Song, Cheng Li, Marie-Laure Fauconnier, Dequan Zhang, Minghui Gu, Li Chen, Yaoxin Lin, Songlei Wang, Xiaochun Zheng

**Affiliations:** a Institute of Food Science and Technology, Chinese Academy of Agricultural Sciences, Key Laboratory of Agro-products Processing, Ministry of Agriculture and Rural Affairs, Beijing 100193, China; b Laboratory of Chemistry of Natural Molecules, Gembloux Agro-Bio Tech, University of Liege, Passage des déportés 2, B-5030 Gembloux, Belgium; c National Center for Nanoscience and Technology, Beijing, 100081, China; d Department of Food Science and Technology, Ningxia University, Yinchuan 750021, China

**Keywords:** Chilled meat, Freshness indicators, Enzyme-like catalysis, Nanozyme sensing systems

## Abstract

It is important to develop rapid, accurate, and portable technologies for detecting the freshness of chilled meat to meet the current demands of meat industry. This report introduces freshness indicators for monitoring the freshness changes of chilled meat, and systematically analyzes the current status of existing detection technologies which focus on the feasibility of using nanozyme for meat freshness sensing detection. Furthermore, it examines the limitations and foresees the future development trends of utilizing current nanozyme sensing systems in evaluating chilled meat freshness. Harmful chemicals are produced by food spoilage degradation, including biogenic amines, volatile amines, hydrogen sulfide, and xanthine, which have become new freshness indicators to evaluate the freshness of chilled meat. The recognition mechanisms are clarified based on the special chemical reaction with nanozyme or directly inducting the enzyme-like catalytic activity of nanozyme.

## Highlights

• Sources of indicators for evaluating chilled meat freshness were analyzed.
• Enzyme-like properties, catalytic mechanisms, and applications of nanozymes were reviewed.
• Details of nanozyme sensing systems for assessing chilled meat freshness were summarized.
• The application limitations and development trends of nanozyme sensing systems were assessed.

## 1 Introduction

During the process of meat processing, circulation, storage and sale, 0–4 °C is the typical fresh preservation method, which can not only guarantee the safety of meat, but also maintain its flavor and reduce nutrition loss(J. Chen et al., 2022). However, chilled meat still faces the challenges of quality and safety problems caused by spoilage. Microbial spoilage(Shao et al., 2021), enzymatic spoilage, lipid oxidation, and protein oxidation(Bekhit, Holman, Giteru, & Hopkins, 2021; Ghaly, 2011) are four main spoilage forms of chilled meat, and protein oxidation has a significant impact on chilled meat freshness. Proteins and other nitrogenous compounds are highly susceptible to degradation by enzymes and microorganisms and to producing harmful substances(Pfmp, Phdsp, Vc, & Rvta, 2021). Such as volatile amines(Luo, Ho, Brankovan, & Lim, 2021; Vinci & Antonelli, 2002; Zhong et al., 2018), biogenic amines(Bhagavathi Sundaram Sivamaruthi, Kesika, & Chaiyasut, 2021; X. Zhang et al., 2021), organic sulfides(X. Guo et al., 2022; Yuan, Bariya, Fahad, Wu, & Javey, 2020), aldehydes(Duan, Li, Wang, & Lin, 2022), and organic acids(Duan et al., 2022). It is particularly important to strengthen the real-time and efficient detection of chilled meat freshness to ensure its quality and safety.

Nowadays, sensory evaluation(Fu, Wang, Zhang, Zhou, & Liu, 2019), chemical-microbiological measurements(Duan et al., 2022), chromatography(Quan et al., 2016), chromatography-mass spectrometry(Stillwell, Bryant, & Wishnok, 1987), spectroscopy(Keklik, Demirci, & Puri, 2010), electronic nose(Dowlati, Guardia, Dowlati, & Mohtasebi, 2012), and electronic tongue(H. Li et al., 2023) are main existing detection methods. Although these methods enable detection the freshness of chilled meat, they all have limitations. Sensory evaluation methods are subjective and can not be quantified(Fu et al., 2019). Chemical-microbiological measurements are time-consuming and complicated to perform. Chromatography methods usually require specialized personnel and are high-cost. Therefore, researchers urgently need some novel detection methods to achieve portable, rapid, sensitive, low-cost, and high-specificity testing(Mustafa & Andreescu, 2020), which has turned into a research hotpot in chilled meat freshness quality and safety assurance(Erna, Rovina, & Mantihal, 2021).

Nanozyme-based detection method is listed as one of the top ten emerging technologies in chemistry in 2022 for “combining the power of natural and artificial catalysis”(Jiangjiexing, Wang, Zhangping, Sirong, & Zhu, & Qin., 2018; X. Zhang, Lin, Liu, Tan, & Xia, 2020). Nanozymes are nanomaterials with catalytic active sites and mimic the kinetic process of enzymatic reactions(H. Wei & Wang, 2013). They can obtain promising applications in rapid food safety testing with the advantages of high stability, low cost, easy production, and resistance to harsh experimental conditions(Liang & Xiyun., 2019; Lunjie Huang, Hongbin, & Wei, 2019). In the detection process, there are two main recognition mechanisms to realize the qualitative and quantitative detection of the target, including the direct reaction between targets and nanozymes, and the specific reaction between targets and the catalytic activity center of the nanozymes. Nanozyme sensing systems offer affordable, sensitive, specific, user-friendly, and rapid assessment of chilled meat freshness.

Hereby, this paper firstly summarized the main four types spoilage mechanisms of chilled meat, and their degradation products were used as indicators to evaluate the freshness of chilled meat. Secondly, the enzyme-like properties and recognition mechanisms of nanozyme sensing systems and their detection application for evaluating chilled meat freshness were analyzed in this paper. Thirdly, this paper was clearly elucidated two common recognition mechanisms between freshness indicators and nanozymes. At present, although nanozymes-based sensing systems had many superiorities in the biosensor detection field, we had to face some challenges in chilled meat freshness detection. This paper also provided some clues to overcome these problems, and to develop new intelligent, portable, rapid, and real-time efficient freshness detection methods in the future.

## 2 Spoilage mechanisms of chilled meat

### 2.1 Microbial spoilage

**Fig. 1:**
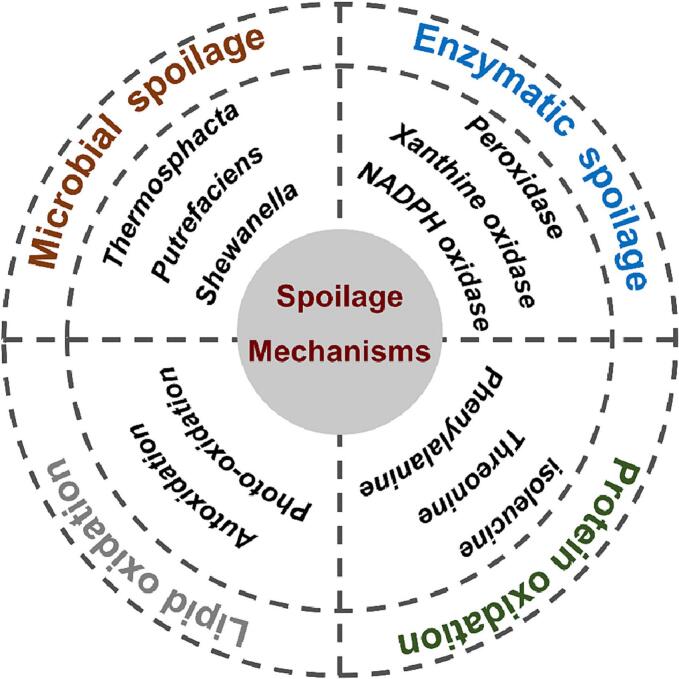
The spoilage mechanisms of chilled meat(Bekhit et al., 2021; Mariutti & Bragagnolo, 2017; Odeyemi & Alegbeleye, 2020; Paczkowski & Schütz, 2011; H. Wang et al., 2017).

The *Aeromonas salmonicida*(H. Wang et al., 2017), *Lactic acid bacteria*(Comi, Andyanto, Manzano, & Iacumin, 2016), and *Serratia, Micrococcus*, *Shewanella*, *Brochothrix* species(X. Zhang, Lin, et al., 2020) are considered as the dominant spoilage bacteria caused by chilled meat (Fig. 1)(Odeyemi & Alegbeleye, 2020; H. Wang et al., 2017), which can make meat odor, discoloration, mucus, and so on(X. Zhang, Lin, et al., 2020). Some studies shown that *Enterococcus casseliflavus* can secrete fat-soluble pigments and produce colored spots on the surface of chilled meat(Tomasevic, Djekic, Furnol, Terjung, & Lorenzo, 2021), and identified the *Vibrio* and *Shewanella* species with high luminescence potential in meat products(Höll, Hilgarth, Geissler, Behr, & Vogel, 2019). Microbial spoilage depends largely on external environmental factors, such as the meat substrate, packaging, temperature, and humidity(X. Chen et al., 2020).

### 2.2 Enzymatic spoilage

Increasing evidence found that many endogenous enzymes in animals play an important role in ensuring the quality of chilled meat after slaughter (Fig. 1)(Bekhit et al., 2021). Calpains improve the tenderness of meat(Bhat, Morton, Mason, & Bekhit, 2018), and aminopeptidases regulate the flavor of meat (Nishimura, 1998). Meanwhile, exogenous proteases produced by bacteria also have similar roles (Jurado, García, Timón, & Carrapiso, 2007). Obviously, the process of enzymatic spoilage has a great influence on the freshness of chilled meat, which not only produce odor, but also produce harmful products. The packaging system, initial microbial composition and count, meat composition, pH value, temperature, and inhibitors can easily influence the process of enzymatic spoilage (Bekhit et al., 2021).

### 2.3 Lipid oxidation

The autooxidation, photo-oxidation, and enzymatic hydrolysis are the three primary oxidation pathways of lipid oxidation in chilled meat. Among them, the first two are of great significance in evaluating the freshness of chilled meat (Fig. 1)(Mariutti & Bragagnolo, 2017). Increasing evidences have proposed that the process of lipid oxidation may be mediated by lipases, lipoxygenases, cyclooxygenases, and several enzymatic systems involved in cholesterol oxidation(Papuc, Goran, Predescu, & Nicorescu, 2017). The progress of lipid oxidation can further induce the protein degradation process, thus leading to the decline of chilled meat quality. Furthermore, adjusting technological parameters in processing effectively inhibits lipoxygenase activity and further prevents the process of lipid oxidation(Jin, Zhang, Yu, Lei, & Wang, 2011).

### 2.4 Protein oxidation

The process of protein oxidation can lead to significant changes in the nutritional quality, physical properties, and sensory properties of chilled meat (Bekhit et al., 2021). Free amino acids (e.g., leucine, isoleucine, threonine, phenylalanine, and tryptophan) are produced by microbial proteases and endogenous proteases through the different degradation pathways of protein, which can be further utilized by decarboxylase to produce common biogenic amines and volatile amines. In addition, sulfur-containing amino acid can be utilized by specific sulfur-containing bacteria to produce hydrogen sulfide(Paczkowski & Schütz, 2011) (Fig. 1). Compared with the other three spoilage mechanisms, the process of protein oxidation is the major causes of spoilage, which can significantly reduce the freshness of chilled meat.

## 3 Freshness indicators of chilled meat

### 3.1 Biogenic amines

**Fig. 2:**
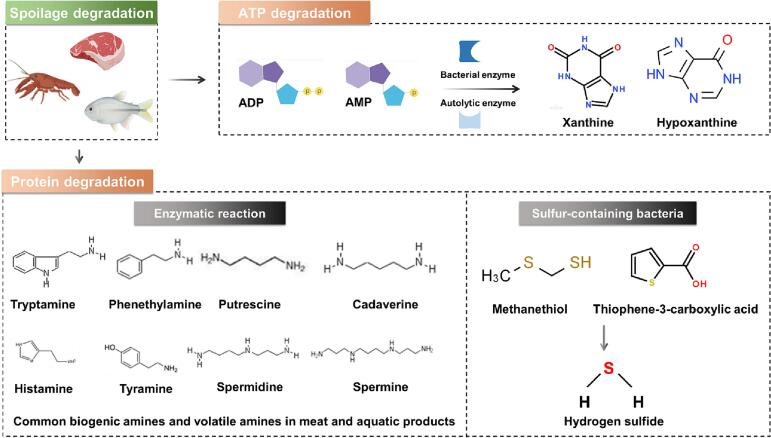
The indicators used to assess chilled meat freshness (Bekhit et al., 2021; Duan et al., 2022; Garg, Singh, Verma, & Monika., 2022; D. Li et al., 2017; Lin et al., 2022; Luo et al., 2021; B. S. Sivamaruthi et al., 2021; Xue et al., 2019; X. Zhang et al., 2021).

Biogenic amines are mainly produced by the enzymatic degradation of free amino acids or by amination and transamination of aldehydes and ketones (Fig. 2)(B. S. Sivamaruthi et al., 2021). According to their chemical structures, biogenic amines can be divided into aliphatic amines (cadaverine, putrescine, spermine, and spermidine), aromatic amines (tyramine and 2-phenylethylamine), and heterocyclic amines (histamine and tryptamine) (Fig. 2) (Duan et al., 2022). Among them, the freshness indicators of tyramine, cadaverine, putrescine, histamine, and trimethylamine are usually used to evaluate the freshness of chilled meat(X. Zhang et al., 2021). A multicolor sensor array, consisting of two types of gold nanostructures (i.e., gold nanorods (Au NRs) and gold nanospheres (Au NSs)), was designed for the identification of spermine (SM), tryptamine (TT), ethylenediamine (EA), tyramine (TR), spermidine (SD), and histamine (HT) in meat(Orouji et al., 2021). The linear range of the multicolor sensor array was 10–800, 20–800, 40–800, 40–800, 60–800, and 80–800 μmol/L. The limit of detection (LOD) was 2.46, 4.79, 8.58, 14.26, 10.03, and 27.29 μmol/L for SD, SM, TT, HT, EA, and TR, respectively.

### 3.2 Volatile amines

During storage and transportation, microorganisms utilize glucose and amino acids to produce volatile amines that can generate obvious odors and reduce the freshness of chilled meat(Hazards, 2016) (Fig. 2). Nowadays, volatile amines serve as a crucial freshness indicator to evaluate the quality of chilled meat (Table 1)(Zhong et al., 2018). For example, a nanofilm was constructed by Zhai and their coworkers to efficiently detect the content of trimethylamine, and its LOD was 0.018 mM(Zhai et al., 2020). Thereafter an intelligent biopolymer film based on starch/carbon nanodots was created, and the visual color of the film changes from purple to green during the storage of pork(Koshy et al., 2021). It can be used as a low-cost portable visual indicator to monitor the freshness of pork.

**Table 1:** Detection of indicators for assessing the freshness of chilled meat.

Freshness indictors	Probes	Systems	Methods	Detection indictors	Food matrices	Linear range	LOD	References
Biogenic amines	AG-AN / GG-2%TiO_2_	Film	Electrochemical	Trimethylamine	Pork; Silver carp	0–0.330 mM	0.018 mM	Zhai et al. (2020)
	Au NPs	Solution	Colorimetric	Dimethyl Sulfide; Histamine	Meat /Fish	0–2.5 μg/mL 0–0.12 μg/mL	0.5 ppm; 0.035 ppm	Chow (2020)
	DPA-Cu NPs	Solution	Fluorescent	Histamine	Pork /Fish	0.05–5 μM	30 nM	X. Zhang et al. (2020)
	Au NRs; Au NSs	Solution	Multicolor sensor array	Spermine; Tryptamine; Ethylenediamine; Tyramine; Spermidine; Histamine	Meat /Fish	10–800; 20–800; 40–800; 40–800; 60–800; 80–800 μmol/L	2.46; 4.79; 8.58; 14.26; 10.03; 27.29 μmol/L	Orouji, Ghasemi, Bigdeli, and Hormozi-Nezhad (2021)
Volatile amines	Carbon nano dots	Film	Colorimetric	Ammonia	Pork	/	/	Koshy, Koshy, Mary, Sadanandhan, and Pothan (2021)
Adenosine triphosphate degradation product	Fe_3_O_4_ NPs	Solution	Electrochemical	Xanthine	Fish	400–2400 μM	4.94 μA/nΜ	Dervisevic and Dervisevic (2019)
	Au NPs	Solution	Electrochemical	Xanthine	Fish, Beef Chicken	1–200 μM	1.4 nA/μM	Dervisevic and Dervisevic (2019)
	SWCNH	Solution	Electrochemical	Hypoxanthine	Fish	1.5–35.4 μM	202.4 mA M^−1^ cm^−2^	Dervisevic and Dervisevic (2019)
	RGO/ZnO	Solution	Electrochemical	Xanthine	Fish	5–400 μM	2.1 μA μM^−1^ cm^−2^	Dervisevic and Dervisevic (2019)
	MWCNT	Solution	Electrochemical	Hypoxanthine	Fish	10–135 μM	1235 nA/μM cm^−2^	Dervisevic and Dervisevic (2019)
	TiO_2_-Gr	Solution	Electrochemical	Hypoxanthine	Meat	20–512 μM	9.5 μM	Albelda, Uzunoglu, Santos, and Stanciu (2017)
Organic sulfides	Cu NCs; CN QDs	Paper-base	Fluorescent	H_2_S gas	Meat	0–3 μM	62.7 nM	X. Huang et al. (2022)
	Ag NPs-BC NCs-MoO_3_ NPs	Film	Colorimetric	H_2_S gas	Meat	24–0.12 μmol/mL	3.27 ppm	Sukhavattanakul and Manuspiya (2021)
	Ag NPs	Hydrogel	Colorimetric	H_2_S gas	Meat	0–15 μM	1.09 μM	Zhai et al. (2019)

### 3.3 Adenosine triphosphate degradation products

The activity of ATP degrading enzyme(Dervisevic & Dervisevic, 2019) gradually increases after slaughter, which can degrade ATP into adenosine diphosphate (ADP), adenosine monophosphate (AMP), and inosine monophosphate (IMP) in turn (Fig. 2)(Garg et al., 2022; Tan, Zhao, Sivashanmugan, Squire, & Wang, 2019). The IMP further degrades into hypoxanthine nucleotide (HxR) through the catalysis of phosphatase and nucleoside phosphorylase(D. Li et al., 2017). Then, hypoxanthine (Hx) and xanthine (XAN) produce by the autolysis and bacterial decomposition of HxR, respectively(X. Gao et al., 2019). The freshness indicators Hx and XAN are more suitable for determining the early spoilage of chilled meat than others (Table 1)(J. Chen, Lu, et al., 2020; C. Guo, You, Li, Chen, & Wang, 2021). An effective electrochemical detection sensor of graphene/titanium dioxide nanocomposite (TiO_2_-Gr) was reported for the detection of Hx(Albelda et al., 2017). The electrochemical sensor showed excellent detection performance for Hx, with a linear range of 20 to 512 μM, the LOD and sensitivity of sensor were 9.5 μM and 4.1 nA/μM, respectively.

### 3.4 Organic sulfides

Sulfur-containing amino acids (e.g., lysine and cysteine) can be decomposed by sulfur-containing bacteria into mercaptans (e.g., thiophenic acid, methanethiol), and further degraded to hydrogen sulfide (H_2_S) (Fig. 2)(Lin et al., 2022). Increasing evidences have proven that H_2_S can be used as an important indicator to evaluate the freshness of chilled meat (Table 1) (Yuan et al., 2020). For example, a H_2_S in situ and nondestructive detection sensor based on gellan gum-capped silver nanoparticles was developed to monitor the meat spoilage process in real time. The LOD of sensor was 1.09 μM, which showed a good selectivity towards H_2_S(Zhai et al., 2019). Then, a novel nanocomposite film label (AgNPs-BCNCs-MoO_3_NPs) was developed for the detection of H_2_S gas in a 1% *w*/*v* solution system(Sukhavattanakul & Manuspiya, 2021). The LOD and limit of quantitation (LOQ) of film label for H_2_S detection were 3.27 and 10.94 ppm, respectively.

## 4 Properties and mechanisms of nanozyme sensing systems

### 4.1 Enzyme-like catalytic properties of nanozymes

#### 4.1.1 Peroxidase-like activity

Peroxidases (POD) use H_2_O_2_ to generate hydroxyl radical (•OH) and further oxidize substrates for a redox reaction(Yan et al., 2019). The inorganic Fe_3_O_4_ nanomaterials can mimic the POD-like catalytic activity reported for the first time in 2007 (L. Gao et al., 2007). Subsequently, precious metal nanomaterials (e.g., Au, Ag, Pt, and Pd), metal oxide/sulfide nanomaterials (e.g., Fe_3_O_4_, Fe_2_O_3_, CoFe_2_O_4_, MnFe_2_O_4_, and ZnFe_2_O_4_), carbon-based nanomaterials (e.g., C_60_[C(COOH)_2_]_2_, Co-g-C_3_N_4_, and Fe-g-C_3_N_4_), and metal-organic frameworks (e.g., MIL-53 (Fe), MIL-101, Fe-MIL-88NH_2_, Cu-MOFs, Co-MOFs, and Co/2Fe-MOFs) were investigated that can mimic the POD-like catalytic activity (Duan et al., 2022). Meanwhile, most nanozymes have lower *K*_m_ value and higher *v*_max_ value than natural peroxidases, which indicate that they have stronger catalytic properties (Table 2) (Ye et al., 2017).

**Table 2:** Enzyme-like type and catalytic performance of nanozymes.

Enzyme-like	Enzyme type	Materials	[E] (M)	Substrate	SA (U mg^−1^)	*K*_m_ (mM)	*v*_max_ (M s^−1^)	*K*_cat_ (s^−1^)	References
Peroxidases-like	Non-SAzymes	Fe_3_O_4_ NPs	2.1 × 10^−12^	TMB	5.143	0.2411	6.55 × 10^−7^	3.1 × 10^5^	Jiang et al. (2018)
		Au NPs	3.4 × 10^−10^	TMB	1.633	0.1277	2.75 × 10^−7^	8.1 × 10^2^	Jiang et al. (2018)
		HRP	6.2 × 10^−11^	TMB	/	0.4376	1.38 × 10^−6^	2.23 × 10^4^	Jiang et al. (2018)
		Carbon NPs	2.2 × 10^−11^	TMB	3.362	0.1037	5.62 × 10^−7^	2.6 × 10^4^	Jiang et al. (2018)
	SAzymes	FeN_4_-SAzyme	1.84 × 10^−7^	TMB	33.8	1.07 × 10^−6^	3.607 × 10^−7^	1.97	Ji et al. (2021)
		Fe BNC SAzyme	3.45 × 10^−6^	TMB	15.41	2.22	1.81 × 10^−6^	0.52	Jiao, Xu, Zhang, Wu, and Guo (2020)
		Fe NC-PdNC	2.14 × 10^−3^	TMB	95.68	1.97	1.43 × 10^−5^	6.68	X. Wei, Song, and Song (2022)
		Fe NC	2.14 × 10^−3^	TMB	30.27	1.35	3.1 × 10^−6^	1.45	X. Wei et al. (2022)
Oxidase-like	Non-SAzymes	Au@Pt	5.0 × 10^−12^	TMB	/	0.013	2.5 × 10^−10^	500	He et al. (2011)
		Co_3_O_4_ NPs	1.0 Mg/mL	TMB	/	0.051	3.3 × 10^−8^	/	Qin et al. (2014)
		Tb_4_O_7_ NPs	7.04 × 10^−10^	TMB	/	1.24 × 10^−4^	4.31 × 10^−8^	1.61 × 10^−4^	C. Li et al. (2019)
		NiCo_2_O_4_ Ms	20 μg/mL	TMB	/	0.127	9.99 × 10^−9^	/	Su et al. (2017)
	SAzymes	FeN_5_ SA/CNF	5.37 × 10^−7^	TMB	/	0.148	7.58 × 10^−7^	0.708	L. Huang, Chen, Gan, Wang, and Dong (2019)
		MnN_5_ SA/CNF	1.50 × 10^−7^	TMB	/	0.253	4 × 10^−7^	0.374	L. Huang et al. (2019)
		CoN_5_ SA/CNF	0.31 × 10^−7^	TMB	/	0.682	1.77 × 10^−7^	0.174	L. Huang et al. (2019)
		FeN_4_ SA/CNF	0.19 × 10^−8^	TMB	/	0.143	4.5 × 10^−8^	0.042	L. Huang et al. (2019)
		NiN_5_ SA/CNF	3.6 × 10^−13^	TMB	/	0.120	6 × 10^−10^	0.0006	L. Huang et al. (2019)
		CuN_5_ SA/CNF	2.35 × 10^−13^	TMB	/	0.124	4.7 × 10^−10^	0.0005	L. Huang et al. (2019)
Catalase-like	Non-SAzymes	N-nanozyme	10 μg/mL	H_2_O_2_	/	360	1.13 mg/L min	/	Juqun Xi et al. (2020)
		Pero-nanozysome	10 μg/mL	H_2_O_2_	/	90	2.34 mg/L min	/	Juqun Xi et al., 2020
		Co_3_O_4_ nanozyme	2 × 10^−4^	H_2_O_2_	/	38.7	3.57 × 10^−5^	0.179	Y. Chen et al. (2023)
	SAzymes	Co-N_4_ SAzyme	1 × 10^−5^	H_2_O_2_	/	24.0	3.72 × 10^−5^	37.2	Y. Chen et al. (2023)
		Co-N_3_P SAzyme	4 × 10^−7^	H_2_O_2_	/	31.8	3.15 × 10^−5^	78.8	Y. Chen et al. (2023)
		Co-N_3_PS SAzyme	1 × 10^−7^	H_2_O_2_	/	6.10	5.20 × 10^−5^	520	Y. Chen et al. (2023)
Superoxide dismutase-like	Non-SAzymes	Fe_3_O_4_ NPs	/	WST-1 by Dojindo	5.6	/	/	/	Cui Liu et al. (2023)
		MnPS_3_	/	WST-1 by Dojindo	721	/	/	/	Cui Liu et al. (2023)
		Pero-nanozysome	/	WST-1 by Dojindo	1257	/	/	/	Cui Liu et al. (2023)
		C-dot SOD nanozyme	/	WST-1 by Dojindo	10,767	/	/	/	Cui Liu et al. (2023)
		Fluorescent C-dot SOD	/	WST-1 by Dojindo	4049	/	/	/	Cui Liu et al. (2023)
	SAzymes	Cu-SAzyme	/	WST-1 by Dojindo	449	/	/	/	Cui Liu et al. (2023)
Glutathione peroxidase-like	Non-SAzymes	MNw	/	H_2_O_2_	/	5.61	0.84	/	Ghosh, Prasad, and Mugesh (2019)
		MNw	/	GSH	/	3.06	0.323	/	Ghosh et al. (2019)
		VNw	/	H_2_O_2_	/	0.04	0.192	/	Ghosh et al. (2019)
		VNw	/	GSH	/	1.28	0.279	/	Ghosh et al. (2019)
Hydrolase-like	Non-SAzymes	OPH	/	Methyl parathion	/	0.21	3.5 × 10^5^	/	Tong et al. (2021)
		VBDA-MIP nano capsule	/	Methyl parathion	/	0.6	8.53.5 × 10^−6^	/	Tong et al. (2021)
		NIP-VBTN	/	Methyl parathion	/	/	1.48 × 10^−6^	/	Tong et al. (2021)
		NIP-VBTNOH	/	Methyl parathion	/	/	3.08 × 10^−6^	/	Tong et al., 2021
		MIP-VBTN	/	Methyl parathion	/	0.85	8.83 × 10^−6^	/	Tong et al. (2021)

([E] is the enzyme or nanozyme concentration. *K*_m_ is the Michaelis constant, *ν*_max_ is the maximal reaction velocity and *K*_cat_ is the catalytic constant, where *K*_cat_ = *ν*_max_/[E] and the *K*_cat_/ *K*_m_ value indicates the catalytic efficiency of the enzyme or nanozymes.)

#### 4.1.2 Oxidase-like activity

Oxidases (OXD) use oxygen to produce reactive oxygen species and further oxidize substrate for a redox reaction(Yan et al., 2019). It has been found that a variety of metal-based and metal oxide-based inorganic nanomaterials (e.g., Ru, Au@Pt, CeO_2_, and N-CNMs) can mimic the OXD-like catalytic activity (Table 2)(Ding, Wang, Sun, & Lin, 2018; Wu & Wang, 2019). Many researches have proved that the formation of intermediates (e.g., singlet oxygen, oxygen, and superoxide anion) and the process of electron transfer greatly affect the OXD-like catalytic properties (C. Liu, Sang, & Yu, 2021). The OXD-like catalytic properties of nanozymes also exhibit through the value of *K*_m_ and *v*_max_, and usually show stronger catalytic activity than natural enzymes. However, the specific catalytic mechanism of OXD-like is unclear.

#### 4.1.3 Catalase-like activity

Catalases (CAT) are a kind of binding enzymes with iron porphyrin as a cofactor, which can decompose H_2_O_2_ to produce O_2_ and H_2_O(Yan et al., 2019). The CAT-like catalytic properties of nanozymes are closely related to the morphology, surface potency, and pH value (Table 2)(Wy et al., 2021). And then, the optimum pH value of CAT-like catalytic is alkaline environment, which is different from POD-like and OXD-like catalytic. Currently, CAT-like catalytic mechanisms mainly involve adsorption activation and redox reactions(Wy et al., 2021). Meanwhile, the intermediate products OH* (* referring to species adsorbed to the metal surface) and •OH play a crucial role in the catalytic process(J. Li, Liu, Wu, & Gao, 2015).

#### 4.1.4 Superoxide dismutase-like activity

Superoxide dismutase (SOD) can catalyze the superoxide anion radical to produce O_2_ and H_2_O_2_ through the electron gain and loss(Yan et al., 2019). Nowadays, Pd, MnO_2_, PB, and fullerene nanomaterials have been reported to have excellent SOD-like catalytic properties (Table 2)(Wy et al., 2021). The chemical structure and surface ions of nanozymes jointly determine their SOD-like catalytic properties, and the optimum pH value is similar to CAT-like catalytic properties. The adsorption activation and electron transfer on the surface of the nanozymes are the two main SOD-like catalytic mechanisms.

#### 4.1.5 Glutathione peroxidase-like activity

Glutathione peroxidase (GP_x_) is an important peroxide-degrading enzyme with selenocysteine as its active center. Nicotinamide adenine dinucleotide phosphate (NADPH) can oxidize glutathione (GSSG) to generate glutathione (GSH) and promote the decomposition of H_2_O_2_ into H_2_O. The GP_x_-like catalytic activity of nanozymes can be obtained by calculating the reduction content of NADPH(Yan et al., 2019). Currently, some nanomaterials (e.g., vanadium metal) have been shown that can mimic the catalytic activity of GP_x_-like (Table 2). The oxidation groups amount on the materials surface and the reaction process of H_2_O_2_ to form peroxide bonds are two main reasons for inducing the catalytic activity of GP_x_-like.

#### 4.1.6 Hydrolase-like activity

Increasing studies have proposed that the catalytic mechanism of hydrolase-like (Hyd-like) is closely related to the breaking of chemical bonds and the generation of free radicals(Wy et al., 2021). The CdTe nanoparticles modified by chiral cysteine that can mimic the catalytic properties of restriction endonuclease and specifically recognize and cleave restriction sites (Sun et al., 2018). Moreover, the chiral copper sulfide quantum dots (d/l-QDs) can mimic the catalytic activity of Hyd-like and cause to the cleavage of peptide bonds between amino acids(Hao et al., 2019). There is little research on the field of food safety detection by using the catalytic activity of Hyd-like.

### 4.2 Catalytic mechanism of nanozymes

#### 4.2.1 Mimicking the enzymatic microenvironment

**Fig. 3:**
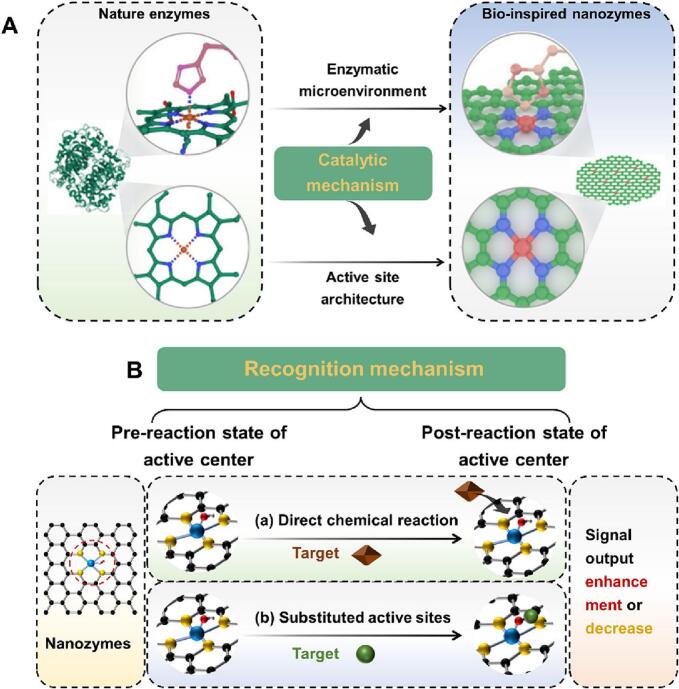
The catalytic and recognition mechanism of nanozymes. A. Catalytic mechanism of nanozymes (Z. Chen, Vorobyeva, Mitchell, & Fako, 2018; C. P. Liu et al., 2016; J. C. Liu & Xiao, 2020; Z. Wang, Zhang, Yan, & Fan, 2020; Xu, Wang, Wang, & Gao, 2019). B. Recognition mechanism of catalytic reaction between the targets and nanozymes (J. Chen et al., 2022; Song et al., 2022; Song et al., 2022).

Nanozymes have been reported to have multi-species enzyme catalytic properties by mimicking the microenvironment of natural enzyme catalysis (Fig. 3A)(Z. Wang et al., 2020). For example, the construct of natural horseradish peroxidase consists of a heme center and a polypeptide chain, which sever as the active site and active site microenvironment, respectively. Among them, the hydrophilic histidine (His) residues in the polypeptide are involved in the localization of H_2_O_2_. The specific catalytic process includes two main steps, the hydrogen bonding enters active site cavity, and further promotes the O—O bond cleavage to form Fe^4+^ = O(C. P. Liu et al., 2016). It has been shown that a novel histidine-functionalized graphene quantum dot (His-GQD)/hemin complex can mimic the catalytic microenvironment of natural horseradish peroxidase for enzyme-like catalytic. Moreover, the AC@O group, O@CAOA group, and GQD group can mimic the heme active site, His ligand, and hydrophobic binding residues of natural horseradish peroxidase, respectively(C. P. Liu et al., 2016).

#### 4.2.2 Mimicking the enzymatic active site architecture

The existence of metal atoms in the structure of nanozymes as the active site provides an important role in its enzyme-like activity (Fig. 3A)(Z. Wang et al., 2020). With the development of nanotechnology, the synthesis of nanozymes with high catalytic activity and strong selectivity will effectively promote the development of food safety detection methods(Z. Chen et al., 2018; J. C. Liu & Xiao, 2020). A type of M-N-C (M = Fe, Co, Mn, etc.) nanozyme is synthesis that can mimic the catalytic properties of natural enzyme. This design can reduce the phenomenon of easy aggregation and uneven distribution of metal atoms, and further improve the metal utilization rate and catalytic activity. Subsequently, a nanozyme (labeled as PMCS) with a remarkable POD-like catalytic activity was synthesized by using a metal-organic framework as a precursor, and the high catalytic property of PMCS is derived from the uniform distribution of single metal zinc atoms(Xu et al., 2019).

## 5 Application of nanozyme sensing systems

Currently, nanozyme sensing systems are gradually applied in the field of food safety detection (Table 3). The recognition mechanisms of systems mainly divided into two types(Song, Li, et al., 2022; Song, Zhang, et al., 2022): (1) Constructed a specific chemical reaction between the targets and nanozymes; (2) Regulated the enzyme-like activity of nanozymes in the targets presence (Fig. 3B).

**Table 3:** Application of enzyme-like catalytic activity in freshness detection.

Methods	Nanozymes	Enzyme-like	Indictors	Food matrices	Linear range	Limit of detection	References
Colorimetric	Fe-PDA NPs	POD-like	Hypoxanthine	Meat	5.13–200 μM	1.54 μM	Y. Zhang, Gao, Ye, and Shen (2022)
	Ce NPs	POD-like	Hypoxanthine	Shrimp	6.2–200 μM	35 μM	Zheng et al. (2023)
	Cu NPs	POD-like	Dopamine	Beef	0.05–100 mM	0.13 mM	Swaidan et al. (2021)
	Ag-Au NPs	POD-like	Spermine	/	115–854 nM	0.87 nM	Kuo et al. (2018)
	Rh NPs	POD-like	Xanthine	Meat	/	/	Shuangfei Cai et al. (2018)
	Rh NPs	POD-like	Xanthine	Meat	1–100 μM	<0.75 μM	Choleva, Gatselou, Tsogas, and Giokas (2017)
Fluorescent	Pt NPs	POD-like	Hypoxanthine	Fish, Shrimp, and Squid	8–2500 μM	2.88 μM	J. Chen, Lu, et al. (2020)
Electrochemical	3D porous graphene NPs	POD-like	Xanthine; Hypoxanthine	Fish	0.3–179.9 μM; 0.3–159.9 μM	0.26 μM; 0.18 μM	Zhu et al. (2021)

### 5.1 Colorimetric detection methods

**Fig. 4:**
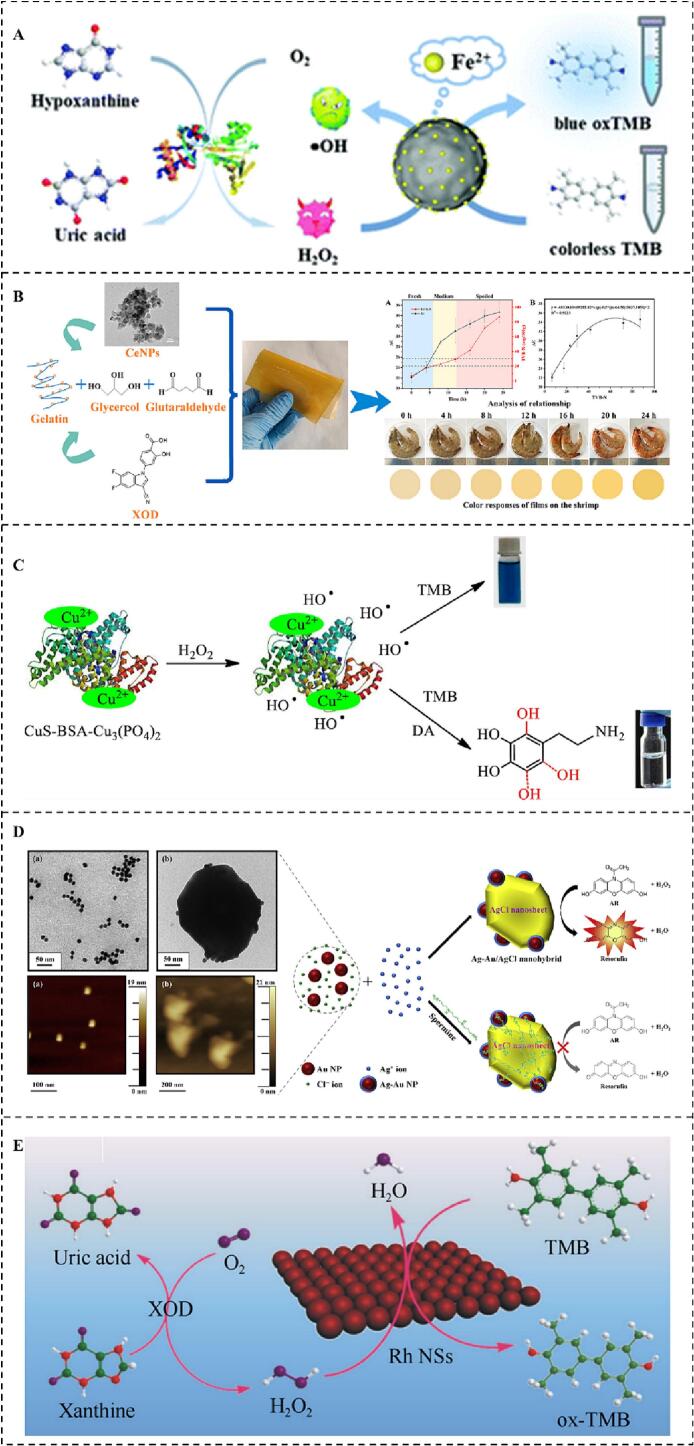
The colorimetric detection methods are applied in detecting chilled meat freshness. A. Hypoxanthine detection(Y. Zhang et al., 2022), B. Hypoxanthine detection(Zheng et al., 2023), C. Dopamine detection (Swaidan et al., 2021), D. Spermine detection(Kuo et al., 2018), E. Xanthine detection(Shuangfei Cai et al., 2018).

The colorimetric detection methods is widely used in rapid detection field because of its advantages of simplicity, portability, low cost, and so on (Duan et al., 2022). For example, an iron-doped polydopamine nanozyme (Fe-PDA) with POD-like catalytic activity was synthesized and used to detect the content of Hx(Y. Zhang et al., 2022). The xanthine oxidase (XOD) catalyzes the reaction of Hx and O_2_ to generate H_2_O_2_, which is further catalyzed by the Fe-PDA nanozyme to produce •OH. The •OH can oxidize the colorless reduced 3,3′,5,5′ -tetramethylbenzidine (_re_TMB) into blue oxidized TMB (_ox_TMB), which can be quantified by measuring the absorbance at 652 nm. The LOD of the Fe-PDA nanozyme-base sensing system was 1.54 μM in the linear range of 5.13–200 μM (Fig. 4A). Similarly, a novel cerium oxide film (Ce NPs) with POD-like catalytic activity was prepared for Hx detection(Zheng et al., 2023). This detection mechanism is similar to the former, and it is also achieved by multi-enzyme cascade catalytic reaction. The LOD of the Ce-NPs-based nanozyme sensing system was 35 μM in the linear range of 6.2–200 μM (Fig. 4B). Then, a nano-sensor (CuS-BSA-Cu_3_ (PO_4_)_2_) with POD-like catalytic activity was constructed for dopamine detection(Swaidan et al., 2021). The presence of dopamine significantly inhibited the POD-like catalytic activity of the CuS-BSA-Cu_3_ (PO_4_)_2_ nanozyme, and the LOD was 0.13 μM in the linear range of 0.05–100 μM. The CuS-BSA-Cu_3_ (PO_4_)_2_ nanozyme sensing system has excellent detection performance in the practical beef samples (Fig. 4C). Meanwhile, a gold‑silver nano complexes nanoprobe (Ag-Au/AgCl) with high OXD-like and POD-like catalytic activity was investigated for spermine detection. The spermine can inhibit OXD-like and POD-like catalytic activity of Ag-Au/AgCl nanozyme(Kuo et al., 2018) (Fig. 4D). And then, a single-atom rhodium nanozyme (Rh SAzyme) with POD-like catalytic activity was prepared for XAN detection. XAN can be oxidized by XOD to produce H_2_O_2_, and the Rh SAzyme uses H_2_O_2_ to exert its POD-like catalytic properties. The LOD of Rh SAzyme-based sensing systems was 0.73 μM in the linear range of 2–80 μM(Shuangfei Cai et al., 2018) (Fig. 4E).

### 5.2 Fluorescent detection methods

**Fig. 5:**
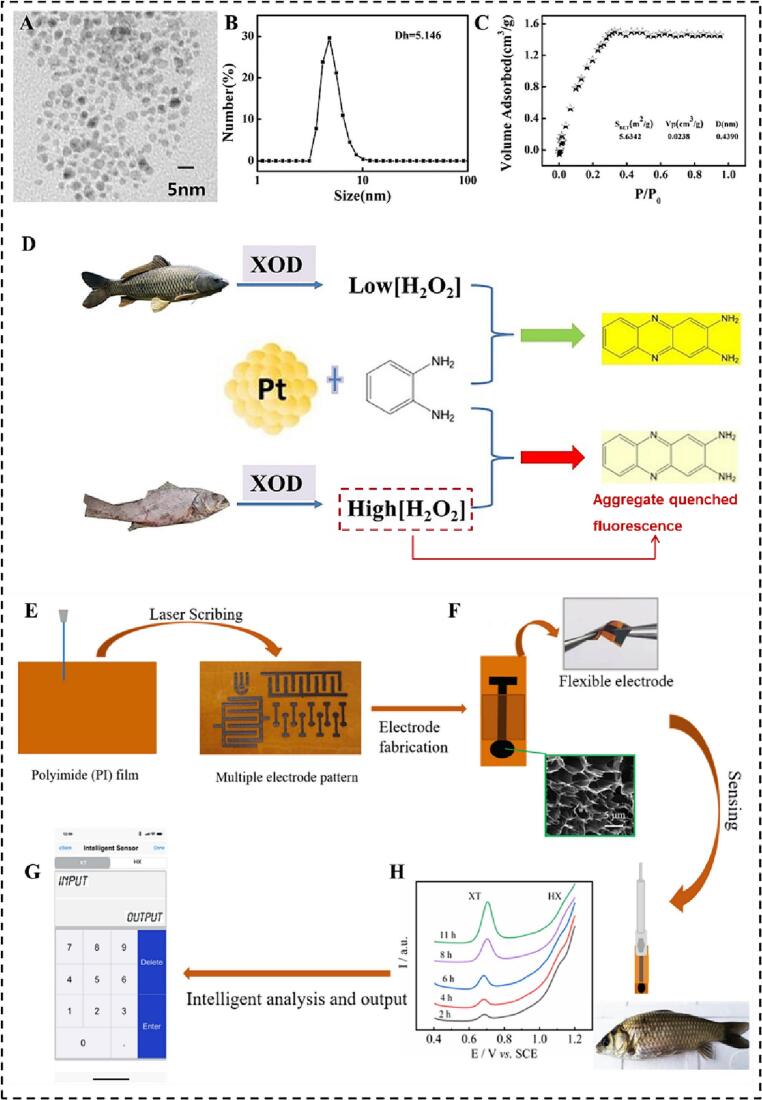
The fluorescent and electrochemical detection method is applied in detecting hypoxanthine(J. Chen, Lu, et al., 2020). A. TEM image, B. DLS analysis, C. N_2_ adsorption-desorption isotherms, D. Principle of detecting aquatic freshness based on the fluorescence biosensor. *E*-H. The electrochemical detection method is applied in xanthine and hypoxanthine detection(Zhu et al., 2021).

Nowadays, fluorescent detection methods play a vital role in the freshness indicators of chilled meat detection. For example, a platinum nanoparticles (Pt NPs) fluorescent biosensor with POD-like catalytic activity was prepared for Hx detection(J. Chen, Lu, et al., 2020). The fluorescence intensity of the Pt-NPs nanozyme sensing system is linearly proportional to the Hx concentration, and the LOD of system was 2.88 μM within the linear range of 8–2500 μM. The Pt NPs used in the sensing system can be reusable, and the recovery rate was 91% after three cycles (Fig. 5A-D). The methods for detecting chilled meat freshness based on the combination of nanozymes and fluorescence materials have the advantage of high sensitivity, velocity, low cost, and portability. However, the use of toxic and hazardous reagents hinders the application of fluorescent detection in food safety detection. The development of eco-friendly fluorescent materials has become one of the effective ways to solve the above problems.

### 5.3 Electrochemical detection methods

Electrochemical detection methods are crucial in the applications of chilled meat freshness detection due to their high sensitivity, rapidity, and portability. At present, voltammetry, amperometry, conductivity, and impedance methods have been reported to be used for detecting the freshness of chilled meat(Johnson, Atkin, Lee, Sell, & Chandra, 2019). For instance, a three-dimensional porous graphene flexible nanozyme electrode with enzyme-like kinetic characteristics was constructed for detecting the freshness indicators of XAN and Hx. The LOD of this system were 0.26 μM and 0.18 μM within the linear range of 0.3–179.9 μM and 0.3–159.9 μM, respectively(Zhu et al., 2021) (Fig. 5E-H). The results provide a better sensing platform for further constructing other electrochemical detection method to assess the freshness of chilled meat. However, although electrochemical detection methods have many excellent catalytic properties for detecting trace targets, the disadvantage of poor stability has limited their practical application.

## 6 Conclusions and perspectives

Chilled meat can be spoiled because of the process of lipid oxidation, enzyme degradation, protein oxidation, and microbial spoilage. Among them, the products of protein and ATP degradation have been as reliable freshness indicators for assessing chilled meat freshness. Such as biogenic amines, hydrogen sulfide, hypoxanthine, volatile amines and so on. These degradation products may directly or indirectly regulate the enzyme-like catalytic properties of nanozymes, which can be achieve specific detection by the means of colorimetric, fluorescent, and electrochemical signal transmission. However, these methods also have some detection drawbacks. Firstly, chilled meat is a complex food matrixes and the spoilage mechanism is also not easy to elucidate, which makes it difficult to ensure the accuracy of detection process. Secondly,

The enzyme-like catalytic properties of nanozymes are unstable and their catalytic activity is still generally low.

Therefore, some measures should be taken to improve these problems existing in the current research. Firstly, the pre-treatment methods of practical samples need to be optimized and the specific freshness indicators need to be filtrated for different species of chilled meat. Secondly, the synthesis methods and conditions of nanozymes need to be improved and optimized, respectively. In this way, it can overcome the problems of catalytic properties. Thirdly, it is necessary to construct a novel cold-adapted nanozyme that can achieve better enzyme-like catalytic properties at cold temperature (0–4 °C). In conclusion, the high enzyme-like catalytic properties of nanozymes occupy a very important position in the field of freshness detection for chilled meat. The nanozyme sensing systems can be made as kinds of rapid detection labels, such as test strip, film, hydrogel, and so on in the future, which have significant advantages to meet current detection needs.

## CRediT authorship contribution statement

**Guangchun Song:** Writing – original draft, Visualization, Investigation, Conceptualization. **Cheng Li**: Writing – review & editing. **Marie-Laure Fauconnier:** Writing – review & editing. **Dequan Zhang:** Writing – review & editing, Conceptualization. **Minghui Gu:** Writing – review & editing. **Li Chen:** Validation, Conceptualization. **Yaoxin Lin:** Writing – review & editing. **Songlei Wang:** Writing – review & editing. **Xiaochun Zheng:** Supervision, Project administration, Funding acquisition.

## Declaration of competing interest

The authors declare that they have no known competing financial interests or personal relationships that could have appeared to influence the work reported in this paper.

## Data Availability

No data was used for the research described in the article.
